# Editorial: Excitotoxicity Turns 50. The Death That Never Dies

**DOI:** 10.3389/fnins.2021.831809

**Published:** 2022-01-17

**Authors:** Alberto Granzotto, John H. Weiss, Stefano L. Sensi

**Affiliations:** ^1^Sue and Bill Gross Stem Cell Research Center, University of California, Irvine, Irvine, CA, United States; ^2^Center for Advanced Sciences and Technology (CAST), University “G. d'Annunzio” of Chieti–Pescara, Chieti, Italy; ^3^Laboratory of Molecular Neurology, Department of Neuroscience, Imaging, and Clinical Sciences (DNISC), University “G. d'Annunzio” of Chieti–Pescara, Chieti, Italy; ^4^Department of Neurology, School of Medicine, University of California, Irvine, Irvine, CA, United States; ^5^Institute for Mind Impairments and Neurological Disorders (IMIND), University of California, Irvine, Irvine, CA, United States

**Keywords:** neuronal death, calcium, zinc, reactive oxygen (nitrogen) species, mitochondria, stroke, ischemia, glutamate

The series of articles of this Research Topic collection celebrates the 50th anniversary of the seminal work published by John W. Olney in 1969 and the pleiotropic properties of glutamate (Olney, 1969).

Glutamate is the primary excitatory neurotransmitter of the Central Nervous System (CNS) and shapes almost every aspect of brain functioning. Olney's landmark study demonstrated, for the first time, that the systemic administration of monosodium glutamate in newborn mice produces a widespread neuronal loss, thereby revealing the “dark side” of the amino acid. The discovery paved the way for a fascinating new field in neuroscience: glutamate-driven neuronal death.

The following decades provided critical insights into the molecular underpinnings of the excitotoxic process. The ionic dependence of excitotoxicity and the crucial role played by the overactivation of the N-methyl-D-aspartate receptors (NMDARs) was revealed by joint efforts of Steve Rothman and Dennis Choi (Choi). The excitotoxic cascade hypothesis emerged as a valid theoretical framework for many neurological conditions like stroke, Alzheimer's disease, Huntington's disease, Parkinson's disease, and amiotrophic lateral sclerosis (Zivin and Choi, 1991).

Excitotoxicity has not unveiled all its secrets. Recent findings identified a new mechanism of NMDAR-mediated excitotoxicity driven by the physical interaction with TRPM4, a transient receptor potential channel (Jones, 2020; Yan et al., 2020). This discovery extends the repertoire of partners-in-crime of NMDARs and prompts novel therapeutic targets.

Knowledge gaps are still present, and refinements of the hypothesis are needed as trials set to target the cascade's upstream mechanisms have so far produced disappointing results and undesirable side effects.

“*The more you know about the past, the better prepared you are for the future.”* The Review articles in this collection provide a comprehensive view of the current state-of-the-art in the excitotoxic field and the historical background that shaped our current knowledge.

The review by Choi offers a historical journey through the landmark discoveries that led to our current understanding of the role of excitotoxicity in brain ischemia. The article dwells on the “early days” and discusses the theoretical pillars of excitotoxicity.

Coyle and Schwarcz outline the path that led to the development of injection methods to reliably and consistently study excitotoxic lesions *in vivo*. The techniques had significant implications also in refining the pharmacology of the excitotoxic process and identifying endogenous molecules like quinolinic acid and kynurenic acid that play a role in neuropathological settings.

Wang and Swanson explore a critical step in the excitotoxic cascade: the generation of reactive oxygen species (ROS) that follows the overactivation of NMDARs. The authors revise the classic mitochondrial-centric view and highlight the role of the plasma membrane-localized enzyme NADPH oxidase as a primary source of ROS in excitotoxic settings. The authors also challenge the concept that calcium influx, specifically through NMDARs, is a mandatory step to induce excitotoxicity and discuss recent findings suggesting that non-ionotropic NMDAR signaling contributes to glutamate-driven neuronal death.

Aizenman et al. summarize the decade-long set of studies focused on the relationship between changes in redox states and excitotoxicity. The review highlights three major findings: (1) the discovery of redox-dependent modulatory sites of NMDAR, (2) the oxidative-driven conversion of 3,4-dihydroxyphenylalanine (DOPA) to the neurotoxic compound 2,4,5-trihydroxyphenylalanine (TOPA) quinone, and (3) the contribution of oxidative stress to the mobilization of toxic amounts of intracellular zinc.

The last two reviews of the series (Granzotto et al.; Kim et al.) further analyze the contribution of zinc dysregulation to the excitotoxic process. Granzotto et al. reconcile different aspects of the excitotoxic cascade and pinpoint intraneuronal zinc mobilization as the missing step in the events linking calcium overload, oxidative stress, and mitochondrial failure. Kim et al. discuss different zinc-dependent neuronal death modalities, suggesting the cation contributes to necrotic and apoptotic pathways. Finally, the paper dwells on the zinc-driven activation of AMPK, an event upstream of caspase activation and an exploitable pharmacological target.

As summarized in this Research Topic, 50 years of basic and clinical research advanced our knowledge in excitotoxic settings. However, a long list of failures in clinical trials tells us that the excitotoxic puzzle is far from being solved. Our current understanding is, in fact, still biased by a neuro-centric view and by the use of sub-optimal preclinical models. An emerging set of findings is finally unveiling the largely neglected contribution of non-neuronal cells to the neurodegenerative process (Barres, 2008). Recent studies are also warning that distinctive features of the human cells of the CNS are poorly recapitulated by rodent models (Bakken et al., 2021). A more holistic approach and more relevant, human-oriented disease models will help excitotoxicity live a second youth.

Let's enjoy the journey.

## Author Contributions

All authors contributed to the Editorial and approved the submitted version.

## Funding

AG was supported by the European Union's Horizon 2020 Research and Innovation Program under the Marie Skłodowska-Curie grant agreement iMIND—No. 841665. SS was supported by research fundings from the Italian Department of Health (RF-2013–02358785 and NET-2011-02346784-1), from the AIRAlzh Onlus (ANCC-COOP), from the Alzheimer's Association–Part the Cloud: Translational Research Funding for Alzheimer's Disease (18PTC-19-602325) and the Alzheimer's Association–GAAIN Exploration to Evaluate Novel Alzheimer's Queries (GEENA-Q-19-596282).

## Conflict of Interest

The authors declare that the research was conducted in the absence of any commercial or financial relationships that could be construed as a potential conflict of interest.

## Publisher's Note

All claims expressed in this article are solely those of the authors and do not necessarily represent those of their affiliated organizations, or those of the publisher, the editors and the reviewers. Any product that may be evaluated in this article, or claim that may be made by its manufacturer, is not guaranteed or endorsed by the publisher.
